# Comparative analysis of a plant pseudoautosomal region (PAR) in *Silene latifolia* with the corresponding *S. vulgaris* autosome

**DOI:** 10.1186/1471-2164-13-226

**Published:** 2012-06-08

**Authors:** Nicolas Blavet, Hana Blavet, Radim Čegan, Niklaus Zemp, Jana Zdanska, Bohuslav Janoušek, Roman Hobza, Alex Widmer

**Affiliations:** 1 Institute of Integrative Biology (IBZ), ETH Zürich, Universitätstrasse 16, Zürich, 8092, Switzerland; 2 Institute of Biophysics, Laboratory of Plant Developmental Genetics, Academy of Sciences of the Czech Republic, v.v.i. Kralovopolska 135, Brno, CZ-61200, Czech Republic; 3 Institute of Experimental Botany, Centre of the Region Haná for Biotechnological and Agricultural Research, Sokolovská 6, Olomouc, CZ-77200, Czech Republic

**Keywords:** BAC library, Pseudoautosomal region, PAR, *Silene latifolia*, Sex chromosome, Evolution

## Abstract

**Background:**

The sex chromosomes of *Silene latifolia* are heteromorphic as in mammals, with females being homogametic (XX) and males heterogametic (XY). While recombination occurs along the entire X chromosome in females, recombination between the X and Y chromosomes in males is restricted to the pseudoautosomal region (PAR). In the few mammals so far studied, PARs are often characterized by elevated recombination and mutation rates and high GC content compared with the rest of the genome. However, PARs have not been studied in plants until now. In this paper we report the construction of a BAC library for *S. latifolia* and the first analysis of a > 100 kb fragment of a *S. latifolia* PAR that we compare to the homologous autosomal region in the closely related gynodioecious species *S. vulgaris*.

**Results:**

Six new sex-linked genes were identified in the *S. latifolia* PAR, together with numerous transposable elements. The same genes were found on the *S. vulgaris* autosomal segment, with no enlargement of the predicted coding sequences in *S. latifolia*. Intergenic regions were on average 1.6 times longer in *S. latifolia* than in *S. vulgaris*, mainly as a consequence of the insertion of transposable elements. The GC content did not differ significantly between the PAR region in *S. latifolia* and the corresponding autosomal region in *S. vulgaris*.

**Conclusions:**

Our results demonstrate the usefulness of the BAC library developed here for the analysis of plant sex chromosomes and indicate that the PAR in the evolutionarily young *S. latifolia* sex chromosomes has diverged from the corresponding autosomal region in the gynodioecious *S. vulgaris* mainly with respect to the insertion of transposable elements. Gene order between the PAR and autosomal region investigated is conserved, and the PAR does not have the high GC content observed in evolutionarily much older mammalian sex chromosomes.

## Background

Heteromorphic sex chromosomes (XY/ZW) can often be distinguished from autosomes by the absence of recombination in at least a part of their length and Y/W chromosome degeneration [1,2]. Plants with sex chromosomes have evolved rarely but repeatedly in many plant lineages, and sex chromosomes have reached various levels of differentiation [3]. In *Asparagus officinalis* and *Carica papaya* for example, X and Y chromosomes have diverged little and recombine along most of their length [4-6] whereas in *Rumex acetosa* and *Silene latifolia,* the sex chromosomes in males are largely non-recombining [7,8]. In *S. latifolia*, X and Y chromosomes can recombine only in the regions known as pseudoautosomal regions (PARs). Westergaard [9] originally identified one PAR on each of the q-arms of *Silene latifolia* sex chromosomes. Later, Lengerova *et al*. (2003) [8] using fluorescent in situ hybridization (FISH) revealed that the X PAR is located on the p-arm, whereas the PAR on the Y chromosome is located on the q-arm. More recently, Scotti and Delph [10] proposed that PARs exist on both ends of the X and Y chromosomes, similar to the situation in humans [11]. A further similarity to mammalian sex chromosomes is that the *S. latifolia* sex chromosomes diverged gradually [12], which led to the formation of evolutionary strata. Comparisons between the evolutionarily young *S. latifolia* sex chromosomes (about 10 million years [12,13]) and those of eutherian mammals (about 110 million years [14]) have revealed that similar processes are involved in the evolution of sex chromosomes in both animals and plants.

The sex chromosomes of *S. latifolia* most likely evolved from a single pair of autosomes as previously shown [12,13], with one autosome of the gynodioecious relative *S. vulgaris*, a species lacking sex chromosomes, carrying homologues of *S. latifolia* sex-linked genes [12,13]. *Silene latifolia* and *S. vulgaris* have the same haploid chromosome number (n = 12), but differ substantially in genome size. The *Silene latifolia* haploid genome is 2646 Mbp in females [15], with the X chromosome being about 400 Mbp in length [16], whereas the haploid genome size of *S. vulgaris* is 1103Mbp [17] and autosomes are about 100 Mbp long.

In this study we analyzed a part of the *S. latifolia* PAR located on the p-arm of the X chromosome and on the q-arm of the Y chromosome (henceforth referred to as PAR) and of the corresponding *S. vulgaris* autosome, in order to study collinearity and divergence between these chromosome parts and to assess whether the *S. latifolia* PAR has characteristics in common with animal PARs. Furthermore, we investigate whether the *S. latifolia* size increase relative to *S. vulgaris* reflects the increase in size of the entire X chromosome or more closely resembles the increase seen in *S. latifolia* autosomes.

In mammalian genomes, PARs have several interesting properties including increased GC content, higher mutation rates and a level of recombination higher than in the rest of the genome [18,19] due to the necessity for crossing over in this region [20]. PARs in mice and the human PAR1 appear to serve a critical function in spermatogenesis, as indicated by the fact that their absence prevents X and Y chromosome segregation during male meiosis, causing male sterility [21-23]. However, PARs differ widely in size among mammals (covering about 4 % of the Y chromosome in humans [24,25] and mice [26], about 8 % in cattle [27] and about 24 % in dogs [28,29]), with most eutherians sharing the same genes situated closest to the telomere but having the pseudoautosomal boundary (PAB), separating the PAR from the sex-specific part of the sex chromosomes, at variable positions [27]. In mice, the PAB is located in the gene *Fxy*. Exons 1–3 are located in the X specific part, while exons 4–10 are located in the PAR [30]. The segment of this gene located in the PAR has a higher GC content than its X-specific portion [30,31].

In order to analyze the *S. latifolia* PAR and the corresponding region on the *S. vulgaris* autosome, we first established and screened a bacterial artificial chromosome (BAC) library of *S. latifolia* with the marker ScOPA09 that has previously been found to be located in the PAR of the closely related dioecious species *S. dioica*[32] and has successfully been identified and used for mapping *S. latifolia* sex chromosomes [12]. The marker ScOPA09 is located in the *S. latifolia* PAR which is known to recombine once per generation in males [33] and makes up about 10 % of the Y chromosome [34,35]. In *S. vulgaris*, the marker OPA is lacking. We therefore first sequenced a clone of the *S. latifolia* BAC library containing the marker ScOPA09. Sequencing was performed by Sanger and 454 pyrosequencing to explore the suitability of different sequencing strategies for BAC assembly. From these sequences we identified new markers and used them to screen the *S. vulgaris* BAC library for a homologous clone. Both BAC sequences were assembled into >100,000 bp-long scaffolds using GS De Novo Assembler (Roche).

Here we present the results of a genomic comparison between an area located in the PAR of the X chromosome p-arm and in the q-arm of the Y chromosome of the dioecious plant species *S. latifolia* and its homologous autosomal area in the closely related gynodioecious species *S. vulgaris*. Our results identify the first physically mapped genes located in the *Silene* PAR and reveal characteristics of a plant pseudoautosomal region.

## Results and discussion

Our study reports the first comparative analysis of a BAC sequence from a plant pseudoautosomal region and the corresponding autosomal area in a related species that lacks sex chromosomes. Comparative mapping of a limited number of sex-linked genes in *S. latifolia* and autosomal genes in *S. vulgaris* has previously demonstrated large-scale synteny between the X chromosome of *S. latifolia* and one *S. vulgaris* autosome [12,13]. Our results provide the first evidence for small-scale synteny and strong collinearity at the gene level within a restricted region of the *S. latifolia* sex-chromosomes, the PAR located in the p-arm of the X chromosome and in the q-arm of the Y chromosome, and the corresponding *S. vulgaris* autosomes.

### BAC sequencing, assembly and annotation

The 454 paired-end sequencing of both a *S. latifolia* BAC clones containing marker ScOPA09 and of a homologous BAC clone from *S. vulgaris* gave more than 150,000 reads for each BAC clone. These were assembled into 171,870 bp and 116,096 bp-long scaffolds for *S. latifolia* and *S. vulgaris*, respectively.

We found a total of twenty-eight homologous sequences (seventeen different accession numbers) with the *A. thaliana* proteome. Of these, nine were found in both *Silene* species, two were identified only in *S. vulgaris* (one of them twice), and six were found only in *S. latifolia*. A total of 16 out of 28 sequences are most likely transposable or repeated elements as indicated by their annotations extracted from the protein domain family database ProDom [36] and repeat coverage (Table 1). The repeat coverage is based on BLAST hits with a *Silene* repeated elements library [37].

**Table 1:** Putative transposable elements identified in *Silene* BAC clones

**Found in**	**TAIR accession**	**Function**	**ProDom annotation**	***Silene* repeats coverage**
*S. latifolia*/ *S. vulgaris*^a^	ATMG00860	Mitochondrion/ hypothetical protein	Transposable element	0 %
*S. latifolia*/ *S. vulgaris*	AT4G23160	Cysteine-rich receptor-like protein kinase 8/ polyprotein	Transposable element	80 %
*S. latifolia*	ATMG00710	Mitochondrion/ hypothetical protein	Transposable element	100 %
*S. vulgaris*^a^	AT3G01410	Putative RNase H	Transposable element	77 %
*S. latifolia*	ATMG00310	Mitochondrion/ hypothetical protein	Transposable element	49 %
*S. latifolia*	AT5G41980	Uncharacterized protein	Transposable element	0 %
*S. latifolia*/ *S. vulgaris*	AT2G01050	Nucleic acid binding / zinc ion binding/ uncharacterized protein	Transposable element	25 %
*S. latifolia*/ *S. vulgaris*	AT1G43760	Uncharacterized protein	Transposable element	20 %
*S. latifolia*	AT4G20520	RNA binding / RNA-directed DNA polymerase/ uncharacterized protein	Transposable element	0 %
*S. latifolia*	ATMG01250	Mitochondrion/ hypothetical protein	Transposable element	23 %

^a^The sequence has been found twice in the BAC clone.

Putative transposable elements were identified by BLASTX searches against the *Arabidopsis thaliana* proteome (TAIR10) with an E-value cut-off of 1E-4, ProDom annotations and coverage analysis with the *Silene* repeated sequences database [37].

Among the nine sequences shared by the two *Silene* species, the areas matching TAIR accessions AT4G23160 (*CRK8*), AT2G01050, AT1G43760 and ATMG00860 contain repeated elements. Moreover, in *S. vulgaris*, the part matching ATMG00860 is contained in a match with the transposon sequence Q3I6J4_SILLA, which also includes the sequence matching accession number AT3G01410 (Table 1). Using annotated *Silene* transposable elements [34] we found that the region matching *CRK8* is similar to a *Copia*-like retrotransposon [38], and that the region matching Q3I6J4_SILLA is similar to a *Retand*-like retrotransposon (see Kejnovský *et al.* (2006) for description [39]). Moreover, the sequences matching AT2G01050, AT1G43760 and *CRK8* are found in both scaffolds at different positions, which provide further evidence that these sequences are transposable elements.

The five remaining sequences correspond to new pseudoautosomal genes. They are homologues of the *A. thaliana* genes *ESP1* (AT4G22970), *BIP1* (AT5G28540), *ACBP1* (AT5G53470), and of genes AT5G53500 and AT5G41970. These latter two genes we named PAR1 and PAR2, respectively (Table 2).

**Table 2:** Putative genes identified in *Silene* BAC clones

**Gene name**	**Found in**	**TAIR accession**	**Putative function**	**ProDom annotation**	**%identity**
*ESP1*	*S. latifolia*/ *S. vulgaris*	AT4G22970	Separase	Separase	34/36
*BIP1*	*S. latifolia*/ *S. vulgaris*	AT5G28540	Luminal-binding protein 1	ATP-binding	80/74
*ACBP1*	*S. latifolia*/ *S. vulgaris*	AT5G53470	Acyl-CoA-binding domain-containing protein 1	Lipid-binding	44/42
PAR1	*S. latifolia*/ *S. vulgaris*	AT5G53500	WD-40 repeat family protein	Hydrolase phosphatase	41/42
PAR2*	*S. latifolia*/ *S. vulgaris*	AT5G41970	Uncharacterized protein	Metal dependent	56/70
PAR3	*S. latifolia*	AT3G15000	Uncharacterized protein	Plastid developmental protein DAG	54/-
SVA1	*S. vulgaris*	AT4G27700	Rhodanese-like domain-containing protein	Rhodanese	-/71

% identity with *A. thaliana* sequence is given first for *S. latifolia* and then for *S. vulgaris*. * PAR2 is truncated approximately by half in *S. latifolia.*

Putative genes were identified using BLASTX searches against the *Arabidopsis thaliana* proteome (TAIR10) with an E-value cut-off of 1E-4, ProDom annotations were used to determine putative function or detect repeats and transposable elements.

Finally, two other gene sequences, AT3G15000 and AT4G27700, that we named PAR3 and SVA1, were found on the BACs of *S. latifolia* and *S. vulgaris*, respectively (Table 2). However, because the BAC sequences only partly overlap, we do not have the homologous copies of these genes in the other species. The PAR3 gene in *S. latifolia* corresponds to a putative pseudoautosomal gene. The *S. vulgaris* SVA1 sequence is homologous to a gene coding for a rhodanese protein in *A. thaliana* (information collected from TAIR [http://arabidopsis.org/]).

Genes located in PARs close to the PAB (less than 50 cM) often present sex-specific expression [40]. Using RNA-seq data from Muyle *et al.*[41] we found no evidence for sex-biased expression of the genes located in the *S. latifolia* PAR ( Additional file 1: Table S1).

### GC content

In mammalian PARs, high recombination associated with biased gene conversion (BGC) [42-44] results in a high GC content [27,30,45,46]. Comparisons of GC and GC3 content between the PAR and non-PAR regions of the human X chromosome revealed a higher GC and GC3 content in the pseudoautosomal region [42]. Further studies of the human PAR revealed that the GC content decreases from 64 % close to the telomeric region to 55 % in the middle of the PAR and is only 38 % close to the pseudoautosomal boundary (PAB) [19]. Similar declines of the GC content were also found in other mammals, including cattle [27] and murine species [47].

A recent analysis of sequence polymorphisms in plants has revealed that the mating system affects GC content, with a higher content in outcrossing compared to selfing taxa being observed, but this effect is significant only in Poaceae that are known to have unusual GC contents [48]. Whether this effect is due to BGC and why it is observed only in Poaceae, however, is not clear. To date, evidence for BGC in plant sex chromosomes is lacking, but given the relatively small size of the investigated PAR (about 10 % of the Y chromosome [34,35]) in *S. latifolia,* which is comparable to PAR size in mammals, and the fact that recombination occurs during meiosis, a higher recombination rate is expected in this region as compared with other regions of the genome.

In contrast to mammals, the *Silene latifolia* PAR can directly be compared with a homologous autosomal region in a closely related species. If the *S. latifolia* PAR has an increased GC content compared to autosomes, then this should be detectable in a comparative analysis. We then determined the GC and GC3 contents for each gene (Table 3). A comparison between *S. latifolia* and *S. vulgaris* revealed no significant difference (GC content: t = 0.0638, p-value = 0.9507; GC3 content: t = 0.0521, p-value = 0.9597). Moreover we determined the GC and the GC3 content of nine sex-linked genes that had previously been identified and are located in the sex-specific region of the X chromosome [49-57] ( Additional file 2: Table S2). No difference was found in the GC and GC3 content between the newly identified PAR genes and the genes located in the sex-specific region of the X chromosome (t = 0.601, p-value = 0.5656 and t = 0.6295, p-value = 0.5437, respectively). These results indicate that the pattern typical for mammalian PARs is not present in the investigated part of the *S. latifolia* PAR. This may indicate that the *S. latifolia* PAR maintains its “autosomal” features, as the sex chromosomes in this species are evolutionarily young. Alternatively, the studied region of the *S. latifolia* PAR might be close to the pseudoautosomal boundary where the GC content is lower than in more distal PAR areas, as has been found to be the case in most mammals [19,27,46]. Indeed, the ScOPA09 marker was estimated to be located 15 cM from the pseudoautosomal boundary (PAB) in *S. dioica*[32]. We obtained a very similar estimate of 11 cM for *S. latifolia* in this study (both calculations were done using Kosambi and Haldane mapping functions, for details see Additional file 3: Table S3). Even though we presently do not know the physical distance between the PAB and the BAC clone studied here, the results of our genetic analysis clearly show that all genes identified in this study are located in the PAR, as evidenced by their cosegregation with marker ScOPA09 ( Additional file 4: Table S4). Furthermore, these genes are recombining with the same recombination frequency of 11 % with the PAB ( Additional file 3: Table S3).

**Table 3:** GC and GC3 content comparison

**Gene name**	***ESP1***	***BIP1***	***ACBP1***	**PAR1**	**PAR2**
Sl sequence length (bp)	5416	2013	1125	2049	507
Sl exon GC content (%)	42.4	47.3	48.3	42.3	48.3
Sl exon GC3 content (%)	41.2	53.5	42.9	38.1	49.7
Sv sequence length (bp)	5416	2013	1125	2049	507
Sv exon GC content (%)	42.4	47.3	47.7	42.4	48.3
Sv exon GC3 content (%)	41.1	53.7	42.9	37.3	49.1
Sl putative gene GC content*	38.2	42.3	38.8	40.4	39.9
Sv putative gene GC content*	37.5	42.8	39.2	40.2	40.6

Sl: *Silene latifolia*, Sv: *S. vulgaris.*

*Exon and intron sequences were taken into account.

GC and GC3 contents were computed on gene sequences of identical size in both *S. latifolia* and *S. vulgaris*. PAR2 is truncated in the BAC sequence of *S. latifolia* and *ESP1* is truncated in *S. vulgaris*. In these cases, only partial sequences with coverage in both species were compared.

### Structure comparison

**Figure 1:**
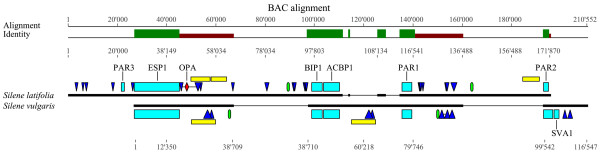
**Alignment of Silene latifolia and S. vulgaris BAC scaffolds.** Genes (light blue), transposable elements (dark blue), full LTR transposon sequences (yellow) and uncharacterized nucleotides (light green) are annotated on the BAC sequences (black bold line). Regions of high identity (green), low identity (dark red) and gaps (fine black line) are indicated. The position of marker ScOPA09, a PAR-specific marker used to identify BAC clones located within the *S. latifolia* PAR, is indicated by a red diamond

Large-scale collinearity between the *S. latifolia* X chromosome and *S. vulgaris* autosome has repeatedly been reported in studies of *S. latifolia* sex chromosome evolution [13,52,58,59]. In addition to large-scale collinearity we here report the presence of small-scale collinearity spanning five genes whose linear arrangement is conserved. We also show that the length of these genes is identical between the studied *Silene* species. Indeed, while we assessed whether the investigated *S. latifolia* PAR presents signs of chromosome enlargement, because the X chromosome is about four times the size of a *S. vulgaris* autosome, we analyzed exon and intron lengths of the five genes previously reported (Table 4). We then compared with a Student’s t-test whether the average size of both introns and exons of the different genes between both species were similar and we found no significant difference between *S. latifolia* and *S. vulgaris* (t = −0.0817, p-value = 0.9369 and t = −0.005, p-value = 0.9961 for intron and exon comparisons respectively). The conserved intron size may indicate a functional role in gene regulation. Indeed, introns enlarged by repetitive elements were found to affect gene expression in rice [60]. However, substantial differences in length occur in intergenic regions due to transposable element insertions. Figure 1 presents the global alignment of both BAC sequences. Intergenic regions are highly diverged in size and consequently major gaps are visible in the alignment. We considered as intergenic all regions in-between the five genes described in this paper for which there are copies in both *Silene* species. The total length of the intergenic region in the *S. latifolia* BAC is 115,909 bp, and 71,866 bp in the *S. vulgaris* BAC. This difference of 44,043 bp is highly significant (χ² = 30823.1, df = 3, p-value < 2.2e-16) and corresponds to a 61 % increase of the *S. latifolia* chromosome size as compared to *S. vulgaris*. This increase is due to the insertion of transposable elements in the *S. latifolia* PAR region.

**Table 4:** Comparison of gene sizes

**Gene name**	***ESP1***	***BIP1***	***ACBP1***	**PAR1**	**PAR2**
Sl sequence length (bp)	15455	3401	4909	3107	1127
Sl exons	24	6	6	7	3
Sl exon length (bp)	5416	2019	1143	2049	510
Sl intron length (bp)	10039	1382	3766	1058	617
Sv sequence length (bp)	16677	3217	5042	3043	1138
Sv exons	24	6	6	7	3
Sv exon length (bp)	5422	2046	1143	2049	507
Sv intron length (bp)	11255	1171	3899	994	631
% identity: entire gene	80.7	85.6	73.6	92.6	92.8
% identity: exons	96.5	96.9	92.9	97.1	95.9
Sv/Sl introns	1.1	0.9	1.0	0.9	1.0

Sl: *Silene latifolia*, Sv: *S. vulgaris.*

Genes PAR2 and ESP1 are truncated in *S. latifolia* and *S. vulgaris* respectively, due to their positions at the end of the investigated BAC clones. For these genes, only the fragment covered in both species was reported below.

### Microsatellite comparison

We found 577 and 377 microsatellite loci in *S. latifolia* and *S. vulgaris* respectively. A comparison of the average proportion of microsatellite loci (mono-, di-, tri- and tetranucleotide microsatellite) between both *Silene* species using a Student’s t-test revealed no significant difference (t = 0.5461, p-value = 0.5867). A previous analysis of microsatellites in plants revealed a negative correlation between microsatellite frequency and genome size [61]. The *Silene latifolia* X chromosome is about four times larger than the *S. vulgaris* autosome. However, our results revealed that the *S. latifolia* PAR contains a similar density of microsatellite repeats as the *S. vulgaris* autosomes, suggesting that microsatellites play no or only a minor role in the size increase of the *Silene latifolia* PAR. Then, we searched for long-mer microsatellite accumulation in *S. latifolia* and *S. vulgaris* (see Methods), which are expected to be rare in the PAR [62]. We found one (ATC)_10_ microsatellite locus in *S. latifolia* and one occurrence each of (T)_35_, (ATA)_16_, (AAG)_19_ and (TTA)_25_ in *S. vulgaris*. The low density of long-mer microsatellites observed here confirms the previously reported paucity of microsatellite repeats on the *S. latifolia* X chromosome PAR [62].

### Transposable element insertion

We found three transposable elements containing long terminal repeats (LTR) in *S. latifolia* and two in *S. vulgaris*. The estimates of the invasion of these elements vary from about 17,200 years ago to 766,000 years ago ( Additional file 5s: Table S5). These elements were inserted after the divergence of the *Silene* sex chromosomes approximately 5 ~ 10 million years ago [12], which may be an indication of highly active transposable elements in the PAR. However, given that we have observed a smaller than expected size increase in the PAR (about 1.6x instead of about 4x), we hypothesize that the enlargement of the X chromosome occurs mainly in the non-PAR areas of the X chromosome and is due to large-scale accumulation of different tandem repeats [63,64] and retrotransposons [65,66]. The observed larger size of the studied *S. latifolia* PAR segment in comparison to the *S. vulgaris* autosome is close to the approximate difference in size between autosomes in *S. latifolia* and *S. vulgaris* and may therefore not reflect the size increase seen in the sex chromosomes.

## Conclusions

In this study we present the first analysis of a fragment belonging to the *S. latifolia* pseudoautosomal region located in the p-arm of the X chromosome and the q-arm of the Y chromosome. The analysis of BAC sequences revealed five new pseudoautosomal genes that are conserved in size and linear arrangement between *S. latifolia* and *S. vulgaris*, indicating small-scale gene collinearity between the X chromosome and the corresponding autosomal region. No increase in GC or GC3 content was found in the studied PAR area, indicating that either the evolutionarily young *S. latifolia* PAR is not GC rich or alternatively, that the studied region is close to the pseudoautosomal boundary, where no increase in GC content is expected. A structural comparison revealed that non-coding regions of the *S. latifolia* PAR contain multiple transposable and repeated elements and are overall about 61 % longer than in *S. vulgaris*. This size increase is similar to the size difference between *S. latifolia* and *S. vulgaris* autosomes and may therefore reflect a genome-wide, rather than a sex chromosome-specific trend. Our study reports the first comparative analysis of a partial pseudoautosomal region in a plant which we compare to a closely related species lacking sex chromosomes, thereby providing new insights into genome size and sex chromosome evolution in *Silene latifolia*.

## Methods

### BAC preparation

*S. latifolia* and *S. vulgaris* were grown from seeds in a climate chamber. Fresh leaf material was harvested after initiation of flowering. *Silene latifolia* leaves were snap frozen in liquid nitrogen, packaged in dry ice and shipped to Amplicon Express, Pullman, Washington, where the BAC library was constructed from high molecular weight (HMW) genomic DNA following the method described by Tao *et al*. (2002) [67]. The *S. vulgaris* BAC library was assembled in the Institute of Experimental Botany of the AS CR Laboratory of Molecular Cytogenetics and Cytometry, Olomouc. In summary, DNA was partly digested with *Hind*III and inserted into the pECBAC1 vector. Ligations were transformed into DH10B *E. coli* cells (Invitrogen) and plated on LB agar containing appropriate concentrations of chloramphenicol, X-gal and IPTG. Clones were robotically selected with a Genomic Solution G3 and transferred into 384 well plates, grown for 18 h, replicated and frozen at −80°C. In order to identify positions and plate numbers of each clone, they were placed on a grid in duplicate on Hybond N + (Amersham, Biosciences) nitrocellulose membranes in a 4x4 pattern. The membranes were incubated and processed as described by Bouzidi *et al*. (2006) [68]. The *S. latifolia* BAC library was arrayed on six membranes of 18,432 colonies and one membrane containing 9,216 clones. The average insert-size of the library is 128 kb. The *S. vulgaris* BAC library was arrayed on three membranes with 18,432 colonies each. The average insert-size of the library was 110 kb [50]. Probes for radioactive hybridizations were labeled with α^32^P using the Prime-It II Random Primer Labelling Kit (Stratagene) according to the manufacturer’s protocol. The presence of the marker in positive BACs was verified by PCR. BAC DNA was isolated with the Large Construct Kit (Qiagen). The *S. latifolia* BAC clone containing the marker ScOPA09 was then sequenced. In order to test which method was most suitable for subsequent BAC assembly, we used three sequencing methods: shotgun Sanger sequencing, 3 kb and 8 kb 454 paired-end pyrosequencing on a GS-FLX machine (Roche). Sanger sequencing was performed by the GATC Biotech laboratory in Konstanz, Germany, and 454 sequencing by the Functional Genomics Center Zurich (FGCZ). As the ScOPA09 marker is not present in *S. vulgaris*, we developed suitable markers from neighboring loci using the *S. latifolia* BAC sequence in order to identify a homologous BAC clone in *S. vulgaris*. Primers used for screening the BAC library are presented in Table 5.

**Table 5:** PCR primers used to amplify fragments of genes located in the *S. latifolia* PAR

**Gene name**	**Forward-primer (5′ – 3′)**	**Reverse-primer (5′ – 3′)**	**Product size (bp)**
*ESP1*	AAATACCCAGCCCGTAGCTT	TGCTCAATACATGCCTCCAG	414
*BIP1*	CGAAAGATGAAGCTCCCAAG	CCCTTCTTGTCCAAACCGTA	990
*ACBP1*	TTAGCCCTGGCAGTCATCTT	AGGAAGTGTGTTCGGTGGAG	252
PAR1	TTTTCCTCAGGCCATAATGC	GGCTACCGAGAACACCATGT	245
PAR2	CTCCAAATTCTCGGGTTCAA	GCTCAAACACTCCACCAACA	176

The annealing temperature for all primer pairs is 60°C.

### Sequencing, assemblies and annotations

Assembly of the initial shotgun sequences of the *S. latifolia* BAC lead to the identification of numerous relatively short contigs because of the presence of multiple repeat regions. We therefore tested two different 454 pair-end sequencing methods (3 kb and 8 kb) in order to overcome these problems. Both approaches provided similar results, but because the 8 kb paired-end sequencing method requires more DNA, we used 3 kb paired-end sequencing for the corresponding *S. vulgaris* BAC clone ( Additional file 6: Table S6).

All 454 sequences were *de novo* assembled using Roche GS De Novo Assembler with 98 % minimum overlap identity and 60 bp as minimum overlap length. We set the expected depth parameter with reference to the estimated size of the BAC (determined by pulse field gel electrophoresis (PFGE)) and the number of reads sequenced. In addition to 454 sequencing and assembly, we used targeted Sanger sequencing to close gaps remaining in the *S. latifolia* scaffold after the assembly process. On *S. vulgaris* we successfully used the Epicentre transposon insertion Kit (EZ1982K) to fill a 987 bp-long gap remaining after assembly. Nevertheless, a few short stretches remain unsequenced after the assembly. Both scaffolds were submitted to GenBank under accession numbers JN574439-JN574440.

The scaffolds were then annotated by similarity using BLASTX [69] with UniProtKB (Swiss-Prot + TrEMBL, 13 July 2010), the *Arabidopsis thaliana* proteome (TAIR10_20100802) and transposable elements (TAIR9_TE) http://www.arabidopsis.org with an E-value cut-off of 1E-4. We used the exon prediction tool Genscan to identify coding sequences with *A. thaliana* as the training model [70] and used ProDom [36] and a *Silene* repeated element database [37] to detect *Silene*-specific repeats and transposable elements. Transposable element annotations were then completed using annotated *S. latifolia* repeats [34].

### Genetic analysis

In order to verify the pseudoautosomal location of the BAC clone studied here, we performed segregation analyses using nucleotide polymorphisms in the genes *ESP1*, PAR2, *ACBP1* and ScOPA09. First, PCR products of these genes were amplified and sequenced to look for partially sex-linked restriction polymorphisms segregating in the pseudobackcross population RB1. This population was prepared by crossing a female plant from a Swiss population, with a male plant obtained from a cross between a female of an inbred line, U9 (from Utrecht, kindly provided by Prof. Sarah Grant), and male plant from a Swiss population. Putative restriction polymorphisms (CAPS) were verified by restriction analysis, and genotyping was performed in 76 DNA samples available. For the list of primers and restriction enzymes used, see Additional file 7: Table S7. The observed recombination frequencies were used for the calculation of map distances using the onemap package of R [71] with both the Kosambi and the Haldane mapping functions [72,73].

### Structure comparison

Using BLAST [69] annotation results we searched for genes and transposable elements, also using Perfect Microsatellite Repeat Finder (http://sgdp.iop.kcl.ac.uk/nikammar/repeatfinder.html) with default parameters (minimum number of repeats = 3, minimum repeat unit length = 2 and maximum repeat unit length = 100). Mononucleotide repeats were identified manually. Only mononucleotide microsatellites with a minimum repeat number greater than or equal to 12 were counted. We assessed the frequency of all mono-, di-, tri- and tetranucleotide microsatellites and tested whether a smaller density of these microsatellites in *S. latifolia* than in *S. vulgaris* was associated with the observed genome size difference between species [61]. Furthermore, we estimated the frequency of long-mer microsatellite stretches (mononucleotides ≥ 30 repeats, dinucleotides ≥ 15 repeats and trinucleotides ≥ 10 repeats) in both species. We used a combination of *A. thaliana* annotation and Genscan [70] exon prediction to compare the size of intergenic regions and introns, and we measured the percentage identity of exons and introns and the GC content at third codon positions (GC3). In order to take into account the possibility that a gene was truncated due to its position at the beginning or end of a BAC, the gene fragment for which we have coverage in both species was used in calculations, whereas gaps were excluded. Both the GC and GC3 contents of the PAR genes were then compared with the GC and GC3 contents of nine X-linked genes (*DD44XSlAP3X, SlX1, SlCypX, SlX3, SlX4, SlX7, SlX9* and *SlssX*) located in the non-recombining part of the *S. latifolia* sex chromosomes [49-57]. In order to test whether average GC and GC3 contents differ between species we used Student’s t-tests in R [71].

### Transposable element analysis

We identified LTRs of transposable elements using LTR_Finder [74] set to the default parameters. We then aligned paired LTR sequences and determined the number of mutations (substitutions and insertions/deletions) between them. We estimated the age of the LTR invasion using the following equation: N/(2*L*K), where N is the number of base substitutions between the two LTRs, L is the length of the LTR and K the base substitution per site per year [75]. We used a value of K = 23E-9 as the average substitution rate per site per year [76].

## Authors’ contributions

NB performed data analysis and drafted the manuscript. HB screened the *S. latifolia* BAC library and assisted with writing the manuscript. RC screened the *S. vulgaris* BAC library and helped with transposable element analysis. JZ and BJ performed genetic analyses. BJ also assisted with writing the manuscript. RH participated in the coordination of the study and helped with data analysis, interpretation and drafting of the manuscript. AW conceived the study, coordinated and supervised all stages and helped draft the manuscript. All authors read and approved the final manuscript.

## Author’s information

Roman Hobza and Alex Widmer equal contributors as senior authors.

## Supplementary Material

Additional file 1 Table S1.PAR gene expression. Description: Expression data were extracted from Muyle *et al.*[41]. Click here for file

Additional file 2 Table S2.Comparison of GC and GC3 contents in *Silene latifolia* sex-linked genes. Description: GC and GC3 contents were calculated for known sex-linked genes located in the non-recombining part of the *S. latifolia* X chromosome. CDS sequences were extracted from GenBank. Click here for file

Additional file 3 Table S3.Genetic analyses of recombination events between the PAB and markers ScOPA09, *ESP1*, PAR2 and *ACBP1*. Description: Standard errors are indicated in brackets. Click here for file

Additional file 4 Table S4.Genetic analysis of the linkage between the marker ScOPA09 and each gene *ESP1*, *PAR2* and *ACBP1*. Click here for file

Additional file 5 Table S5.LTR insertion time estimates Description: Positions and characteristics of LTRs (Primer binding site (PBS), polypurine tract (PPT), both 5′- and 3′-LTR size) found using LTR_Finder. Time was computed as described by Liu *et al*. (2008): Nucleotide differences / (2 x LTR length x K ). K: substitutions per site per year = 23E-9. Sl = *S. latifolia*, Sv = *S. vulgaris*. Click here for file

Additional file 6 Table S6.Results of the different BAC sequencing approaches and assemblies. Description: The same *S. latifolia* BAC clone was sequenced by shotgun Sanger sequencing and 454 pyrosequencing with different paired-end libraries. Click here for file

Additional file 7 Table S7.Primers and restriction enzymes used for genetic analysis. Description: The annealing temperature for all primer pairs was 60°C. Click here for file
